# Obstructive Sleep Apnea and Non-Alcoholic Fatty Liver Disease: Is the Liver Another Target?

**DOI:** 10.3389/fneur.2012.00149

**Published:** 2012-10-17

**Authors:** Aibek E. Mirrakhimov, Vsevolod Y. Polotsky

**Affiliations:** ^1^I.K. Akhunbaev Kyrgyz State Medical AcademyBishkek, Kyrgyzstan; ^2^Division of Pulmonary and Critical Care Medicine, Johns Hopkins University School of MedicineBaltimore, MD, USA

**Keywords:** sleep apnea, intermittent hypoxia, non-alcoholic fatty liver disease, non-alcoholic steatohepatitis

## Abstract

Obstructive sleep apnea (OSA) is recurrent obstruction of the upper airway during sleep leading to intermittent hypoxia (IH). OSA has been associated with all components of the metabolic syndrome as well as with non-alcoholic fatty liver disease (NAFLD). NAFLD is a common condition ranging in severity from uncomplicated hepatic steatosis to steatohepatitis (NASH), liver fibrosis, and cirrhosis. The gold standard for the diagnosis and staging of NAFLD is liver biopsy. Obesity and insulin resistance lead to liver steatosis, but the causes of the progression to NASH are not known. Emerging evidence suggests that OSA may play a role in the progression of hepatic steatosis and the development of NASH. Several cross-sectional studies showed that the severity of IH in patients with OSA predicted the severity of NAFLD on liver biopsy. However, neither prospective nor interventional studies with continuous positive airway pressure treatment have been performed. Studies in a mouse model showed that IH causes triglyceride accumulation in the liver and liver injury as well as hepatic inflammation. The mouse model provided insight in the pathogenesis of liver injury showing that (1) IH accelerates the progression of hepatic steatosis by inducing adipose tissue lipolysis and increasing free fatty acids (FFA) flux into the liver; (2) IH up-regulates lipid biosynthetic pathways in the liver; (3) IH induces oxidative stress in the liver; (4) IH up-regulates hypoxia inducible factor 1 alpha and possibly HIF-2 alpha, which may increase hepatic steatosis and induce liver inflammation and fibrosis. However, the role of FFA and different transcription factors in the pathogenesis of IH-induced NAFLD is yet to be established. Thus, multiple lines of evidence suggest that IH of OSA may contribute to the progression of NAFLD but definitive clinical studies and experiments in the mouse model have yet to be done.

## Introduction

Obstructive sleep apnea (OSA) is a common disorder that affects 4–24% of men and 2–9% of women in the United States (Young et al., 1993), but the prevalence of OSA exceeds 30–50% in obese individuals Punjabi et al. (2002), Vgontzas et al. (2000), Young et al. (1993), Tufik et al. (2010), Young et al. (2002). OSA is characterized by recurrent upper airway collapse during sleep resulting in fragmentation of sleep and recurrent oxyhemoglobin desaturations termed chronic intermittent hypoxia (IH; Gastaut et al., 1966). OSA markedly increases mortality and morbidity due to the increased cardiovascular risk (Gami et al., 2005; Yaggi et al., 2005; Marshall et al., 2008; Young et al., 2008; Punjabi et al., 2009). The cardiovascular risk of OSA has been attributed to metabolic dysfunction (Jun and Polotsky, 2009; Lavie and Polotsky, 2009; Drager et al., 2010a). OSA is associated with all manifestations of the metabolic syndrome, including visceral obesity, hypertension, dyslipidemia, and insulin resistance (Levy et al., 2009; Drager et al., 2010b; Sharma et al., 2011; Bonsignore et al., 2012). Recent clinical data suggests that metabolic dysfunction of OSA is associated with nocturnal IH, independent of obesity (Drager et al., 2010b; Bonsignore et al., 2012). Experiments in a mouse model of IH mimicking oxyhemoglobin desaturations in patients with OSA showed that IH induces insulin resistance, glucose intolerance, and dyslipidemia in the absence of obesity (Li et al., 2005b, 2006, 2007; Iiyori et al., 2007; Savransky et al., 2007b), but the effects of IH are particularly severe in the presence of obesity (Polotsky et al., 2003; Drager et al., 2011). Recent studies in healthy human volunteers have also shown that IH induces insulin resistance, independent of obesity (Louis and Punjabi, 2009). Insulin resistance and dyslipidemia induced by OSA can be reversed by continuous positive airway pressure (CPAP; Robinson et al., 2004; Sharma et al., 2011). OSA is associated with another manifestation of metabolic dysfunction, non-alcoholic fatty liver (NAFLD; Tanne et al., 2005; Jouet et al., 2007; Kallwitz et al., 2007; Polotsky et al., 2009). Animal and human data indicate that IH of OSA may contribute to the progression of NAFLD, a common condition with the prevalence between 17 and 33% and the major risk factors being obesity and insulin resistance (Hilden et al., 1977; Nomura et al., 1988; Bellentani et al., 2000; Browning and Horton, 2004; Browning et al., 2004; Clark, 2006; McCullough, 2006). In this article, we will review available clinical evidence on the relationships between OSA and liver disease and examine putative mechanisms linking sleep disordered breathing and nocturnal IH with NAFLD. We will begin with a brief overview of NAFLD.

## NAFLD: Clinical Overview

Non-alcoholic fatty liver disease includes a spectrum of the disease severity, ranging from steatosis without inflammation to non-alcoholic steatohepatitis (NASH) and liver cirrhosis (Day and James, 1998; Browning and Horton, 2004; Diehl, 2005). NASH is a progressive fibrotic disease, in which cirrhosis and liver-related death occur in up to 20 and 12% patients, respectively (McCullough, 2004). Approximately 10–30% patients with hepatic steatosis have histological features of NASH (McCullough, 2006; Tilg and Moschen, 2010). NASH is the most common cause of cryptogenic liver cirrhosis in the U.S. (Caldwell et al., 1999; Clark and Diehl, 2003). NASH has also been implicated in causality of at least 7% of cases of hepatocellular carcinoma (Bugianesi et al., 2002). Multiple treatment options were considered for NASH, but, with a possible exception of a moderate benefit from vitamin E, effective therapy is still lacking (Kashi et al., 2008; Torres and Harrison, 2008; Vuppalanchi and Chalasani, 2009; Sanyal et al., 2010).

To this date, liver biopsy remains the gold standard to diagnose and stage NAFLD (Clark and Diehl, 2003; Vuppalanchi and Chalasani, 2009). The Pathology Committee of the NASH Clinical Research Network developed a histological scoring system that determines the severity of NAFLD based on a NAFLD activity score (NAS; Kleiner et al., 2005), which is now being uniformly used. This scoring system assesses a degree of steatosis, lobular inflammation, hepatocellular ballooning, and fibrosis. NAS is a sum of steatosis, lobular inflammation, and hepatocellular ballooning scores. Fibrosis was not included as a component of NAS, because it is less reversible and does not reflect the acuteness of the inflammatory process. NAS ≥ 5 is considered NASH, whereas NAS < 3 excludes NASH (Kleiner et al., 2005).

Liver biopsy is an invasive procedure, therefore non-invasive biomarkers of NAFLD is a desirable alternative. Serum alanine and aspartate aminotransferases (ALT and AST) indicate liver injury, but are neither sensitive nor specific to diagnose NAFLD and characterize its severity (Clark and Diehl, 2003; Browning et al., 2004). Serum alkaline phosphatase (AP) and gamma glutamyl transpeptidase (GGT) may indicate intrahepatic cholestasis, but cannot be used to assess liver injury. Serum ferritin has been suggested as a biomarker of NAFLD (Licata et al., 2009; Manousou et al., 2011; Kim et al., 2012; Kowdley et al., 2012). Serrum ferritin is independently associated with advanced hepatic fibrosis (odds ratio, OR, 1.66) and increased NAS (OR, 1.99; Kowdley et al., 2012). The NASH Clinical Research Network currently recommends serum ferritin to identify NAFLD patients at risk for NASH and advanced fibrosis However, the sensitivity and specificity of ferritin for the diagnosis of NASH are relatively low (Kowdley et al., 2012).

Over the last decade several biochemical panels were developed and tested as surrogate markers of NAFLD. FibroTest includes α2-macroglobulin, apolipoprotein A1, haptoglobin, total bilirubin, and GGT adjusted for age and gender. ActiTest includes same five components and ALT. SteatoTest and NashTest include the same components as ActiTest plus serum glucose, triglycerides, and cholesterol adjusted for age, gender, and BMI (Poynard et al., 2012). The value of these biochemical panels has been systematically reviewed in a recent meta-analysis in severely obese individuals (Poynard et al., 2012). FibroTest has very low sensitivity and negative predictive value for the diagnosis of liver fibrosis (13.4 and 51.5%, respectively), but high specificity and positive predictive value (97.5 and 85%, respectively). Depending on the value cut-off, SteatoTest and NashTest had either low sensitivity and relatively high specificity or vice versa for respective diagnoses of hepatic steatosis and NASH (Poynard et al., 2012). Other biomarker panels such as FibroSpect II (hyaluronic acid, tissue inhibitor of matrix metalloproteinase I, α2-macroglobulin), N-terminal propeptide of type III collagen, and others have not been validated in NAFLD (Baranova et al., 2011). Overall, biochemical serum testing may identify patients at risk of NAFLD and NASH but does not allow reliably stage the disease.

Recent progress in imaging technology, ultrasound, computer tomography, magnetic resonance imaging (MRI), and magnetic resonance spectroscopy (MRS) allow reliably diagnose and quantify hepatic steatosis (Springer et al., 2010; Hernaez et al., 2011; Patel et al., 2011). A novel ultrasound technology FibroScan has recently been developed (Friedrich-Rust et al., 2010; Myers et al., 2012a,b). The FibroScan (Echosens, Paris, France) allows simultaneous assessment of hepatic steatosis and fibrosis. The Controlled Attenuation Parameter (CAP) measures the degree of ultrasound attenuation by hepatic fat simultaneously with liver stiffness measurements. CAP values significantly correlated with the degree of steatosis on liver biopsy (*r* = 0.47–0.51; Myers et al., 2012a). Compared to liver histology, the diagnostic accuracy of ultrasound transient elastography in significant liver fibrosis is 0.80 for the M probe and 0.82 for the XL probe and in liver cirrhosis is 0.91 for the M probe and 0.95 for the XL probe (Friedrich-Rust et al., 2010). The diagnostic performance of the XL probe was found to be superior to the M probe for liver stiffness measurements in obese patients (Friedrich-Rust et al., 2010). Magnetic resonance-based elastography is another promising technique to detect liver fibrosis (Bonekamp et al., 2009). Thus, non-invasive novel imaging techniques can detect hepatic steatosis and liver fibrosis with a reasonable accuracy, but do not allow the assessment of hepatic inflammation.

## NAFLD: Pathogenesis

Day and James (1998) proposed a “two-hit” model to explain the progression of NAFLD. The “first hit” involves the accumulation of triglyceride in hepatocytes, and has been specifically attributed to insulin resistance and obesity. Obesity and insulin resistance are characterized by increased adipocyte mass and increased hormone-sensitive lipase activity, which leads to up-regulation of lipolysis and increased uptake of free fatty acids (FFA) by the liver (Browning and Horton, 2004). In turn, increased FFA uptake induces triglyceride biosynthesis and hepatic steatosis. In addition, high levels of insulin in obese individuals may up-regulate hepatic lipid biosynthesis *de novo* by activating a master-regulator of lipid biosynthesis, a transcription factor sterol regulatory element binding protein-1c (SREBP-1c; Foretz et al., 1999; Shimomura et al., 1999b) and a SREBP-1-regulated enzymes of triglyceride biosynthesis stearoyl coenzyme A desaturase 1 (SCD-1; Cohen et al., 2002; Biddinger et al., 2005) and diacylglycerol acyltransferases (DGAT). Persistent hyperglycemia in patients with diabetes may activate another lipogenic transcription factor, carbohydrate response element binding protein (ChREBP; Iizuka et al., 2004; Dentin et al., 2006; Ma et al., 2006; Postic et al., 2007; Davies et al., 2008). Over-expression of SREBP-1c has been shown to lead to fatty liver in mouse models of insulin resistance and obesity (Shimomura et al., 1998, 1999a), and can account for the development of hepatic steatosis in human subjects. In turn, hepatic fat accumulation increases insulin resistance stimulating gluconeogenesis and activating protein kinase C-ε and janus kinase 1, which interfere with tyrosine phosphorylation of insulin receptor substrates 1 and 2 and impair the ability of insulin to activate glycogen synthase (Samuel et al., 2004; Savage et al., 2007).

When hepatic steatosis progresses to NASH, hepatic lobules become infiltrated with a mixed population of inflammatory cells. Inflammation is followed or accompanied by hepatocyte ballooning and necrosis, appearance of Mallory bodies, and, finally, perisinusoidal fibrosis, or cirrhosis (Ludwig et al., 1997; Carmiel-Haggai et al., 2005). The progression of hepatic steatosis to NASH has been attributed to a “second hit” that leads to the development of liver inflammation and fibrosis (Day and James, 1998). Obesity, age over 45 years, diabetes, hypertriglyceridemia, and hypertension have been identified as risk factors for the progression of NAFLD (Angulo et al., 1999; Dixon et al., 2001; Bugianesi et al., 2002). The progression of NAFLD to NASH was linked to oxidative stress and lipid peroxidation in the liver, leading to inflammation (Robertson et al., 2001; George et al., 2003; Bergamini et al., 2004; Browning and Horton, 2004; Laurent et al., 2004; Carmiel-Haggai et al., 2005; Oliveira et al., 2005; Seki et al., 2005; McCullough, 2006).

“A two-hit” model of NAFLD progression has been recently revised. Simple steatosis, which never progresses in 70–90% of NAFLD patients, might be a separate entity from relentlessly progressing NASH. Inhibition of DGAT-1 or SCD-1 improves liver steatosis but worsens liver damage suggesting that accumulation of triglycerides in the liver may protect against NASH (Yamaguchi et al., 2007, 2008; Li et al., 2009; Tilg and Moschen, 2010). NASH could be a result of multiple hits to the liver with major contribution of pathologically enhanced lipolysis of visceral and subcutaneous adipose tissue and ensuing hepatic lipotoxicity (Neuschwander-Tetri, 2010). Other hits include pro-inflammatory cytokines and adipokines derived from adipose tissues such as leptin, IL-6, TNF-α as well as gut-derived factors and endoplasmic reticulum stress (Tilg and Moschen, 2010). Recent clinical evidence suggests that OSA may lead to NAFLD progression, whereas experimental literature showed that IH induces liver injury and steatohepatitis.

## NAFLD and OSA: Clinical Evidence

Several cross-sectional studies examined levels of liver enzymes in serum of patients with OSA (Table 1). Chin et al. (2003) were first to report abnormally elevated morning AST levels in 14 out of 40 studied OSA patients (35%). Increased liver enzymes correlated with insulin resistance measured by the homeostasis model assessment method (HOMA). Norman et al. (2008) demonstrated in 109 patients with OSA that ALT and AST levels directly correlated with the severity of nocturnal hypoxia, but not with the apnea-hypopnea index (AHI) or BMI. Increased ALT, AST, and AP in adult patients with moderate and severe OSA have also been reported by Shpirer et al. (2010). Gude et al. (2009) studied a randomly selected a population sample of 220 individuals and found that serum GGT levels directly correlated with a degree of nocturnal hypoxemia (Tables 1 and 2). Unfortunately, all of the above studies lacked a control group.

**Table 1:** **Studies that measured liver enzymes and/or imaged liver in patients with OSA**.

Study	Sample size (OSA/controls)	Type of the study	Outcome	Findings
Chin et al. (2003)	Adults 40/0	Cross-sectional followed by a non-randomized trial of CPAP for one night	ALT, AST, TG, insulin, IR	↑ALT and AST in the morning attenuated by CPAP. No change in IR, TG, and insulin
Norman et al. (2008)	Adults 109/0	Cross-sectional study	ALT, AST, glucose, TC, TG, HDL, LDL	Minimal nocturnal SpO2 inversely correlated and % T90 directly correlated with ALT and AST. No effect of AHI or other metabolic biomarkers
Kheirandish-Gozal et al. (2008)	Children 343 patients; 175 non-OSA habitual snorers	Cross-sectional study	ALT, AST, glucose, insulin, TG, HDL, LDL	↑ALT, insulin, TC, LDL, and ↓HDL in patients with OSA, ↑prevalence of OSA in obese patients with elevated ALT
Kohler et al. (2009)	Adults 94/0	Randomized placebo controlled trial of CPAP for 4 weeks	ALT, AST	↓ALT in both therapeutic and sham CPAP groups
Gude et al. (2009)	Adults 220 (only 70 had PSG)	Cross-sectional population study	GGT, glucose, TG, IL-6, TNF-α	GGT inversely correlated with mean and minimal SpO2 and directly correlated with % T90, independent of all other variables. No effect of AHI
Tatsumi and Saibara (2005)	83/41	Cross-sectional study	Liver/spleen ratio by computer tomography, serum P-III-P	P-III-P levels inversely with average nocturnal SpO_2_. No effect of OSA on the liver/spleen ratio
Shpirer et al. (2010)	47/0 CPAP in 16/0	Cross-sectional study and retrospective analysis of CPAP treatment for 3 years	ALT, AST, AP, liver attenuation index by computer tomography	↑ALT, AST, AP in moderate-severe OSA compared to mild OSA. ↓liver attenuation in CPAP-compliant patients (n = 6) compared to non-compliant
Sivam et al. (2012)	38/0	Randomized placebo controlled cross-over trial of CPAP for 2 months	ALT, AST, AP, MRI/MRS	↓AP, no other changes identified
Hoyos et al. (2012)	65/0	Randomized placebo controlled trial of CPAP for 12 weeks followed by 12 weeks of real CPAP in all	Serum leptin, adiponectin, liver and visceral fat by CT, insulin sensitivity	No change at 12 weeks. Improvement of insulin sensitivity at 24 weeks, but no change in liver or visceral fat

*ALT, alanine aminotransferase; AST, aspartate aminotransferase; AP, alkaline phosphatase; GGT, gamma glutamyl transpeptidase; IR, insulin resistance; MRI/MRS, magnetic resonance imaging & magnetic resonance spectroscopy; SpO_2_, oxyhemoglobin saturation; P-III-P, type III procollagen; %T90, sleep time with SaO_2_ < 90%; TC, total cholesterol; TG, triglycerides*.

Several cross-sectional studies compared OSA patients to control subjects. Kheirandish-Gozal et al. (2008) reported significantly higher serum ALT levels in children with OSA compared to children without OSA. In bariatric population, Kallwitz et al. (2007) and Jouet et al. (2007) reported a higher prevalence of elevated ALT levels in patients with OSA compared to those without OSA. In the sleep clinic population, the presence of severe OSA (AHI > 50) was an independent predictor of elevated liver enzymes (Tanne et al., 2005). Notably, several studies in the bariatric and sleep clinic populations found no relationship between liver enzymes and the presence or severity of OSA (Polotsky et al., 2009; Aron-Wisnewsky et al., 2012). Liver enzymes remained within normal limits, despite significant changes on histopathology. One study reported a direct correlation between a serum marker of liver fibrosis, type III procollagen, and triglyceride and fasting plasma glucose, but not ALT or AST (Tatsumi and Saibara, 2005). The effect of CPAP on ALT and AST level was assessed a non-randomized uncontrolled clinical trial by Chin et al. (2003), who reported that one night of CPAP significantly decreased morning AST levels and abolished morning increases in AST and ALT. However, the only randomized placebo controlled study failed to find any effect of therapeutic CPAP treatment for 4 weeks on liver enzymes compared to sham CPAP (Kohler et al., 2009). Overall, evidence that OSA has an independent effect on liver enzyme levels remains inconclusive and may pertain only to severe OSA with marked nocturnal oxyhemoglobin desaturations. Given that elevated liver enzymes are neither sensitive nor specific markers of NAFLD and NASH (Clark and Diehl, 2003; Browning et al., 2004), studies focusing on liver enzymes could not elucidate relationships between OSA and NAFLD. Other serum biomarkers of NAFLD (NAFLD: Clinical Overview) have not been studied in patients with OSA.

### OSA and liver imaging studies

To our knowledge, there have been only four liver imaging studies in patients with OSA, three of which utilized computer tomography (CT) to assess the degree of hepatic steatosis (Tatsumi and Saibara, 2005; Shpirer et al., 2010; Hoyos et al., 2012) and one study used MRI/MRS. Tatsumi and Saibara (2005) measured the amount of visceral and liver fat in patients with OSA (AHI > 5/h) compared to non-OSA subjects and found that the presence of OSA was associated with increased visceral adiposity, but not with the degree of hepatic steatosis (Table 1). Shpirer et al. (2010) retrospectively analyzed liver CT in 47 patients with OSA and found an association between hepatic steatosis and the severity of OSA. However, patients with severe OSA and hepatic steatosis on CT had a higher BMI and, therefore, the results of the study were confounded by obesity. The effect of CPAP treatment for 3 years was examined in 11 patients. CPAP therapy was associated with a decrease in hepatic steatosis in six compliant patients, despite unchanged BMI, but not in five non-compliant patients. Hoyos et al. (2012) recruited patients with moderate-severe OSA and performed a randomized double-blinded sham controlled trial of CPAP for 12 weeks followed by real CPAP treatment for 12 weeks in all patients. Visceral and liver fat were measured by CT; insulin sensitivity was assessed based on fasting blood glucose and insulin; serum leptin and adiponectin were measured. There was no effect of CPAP on liver steatosis or any metabolic parameters at 12 weeks. Insulin sensitivity improved at 24 weeks, while liver fat and all other biomarkers remained unchanged. Sivam et al. (2012) performed a randomized double-blinded sham controlled trial of CPAP for 8 weeks and reported no effect of CPAP on liver fat or liver enzyme with exception of a mild decline in AP. Imaging studies have not been used to assess liver fibrosis in OSA. Thus, literature on liver imaging in OSA is fragmentary and inconclusive.

### OSA and liver pathology

**Figure 1:**
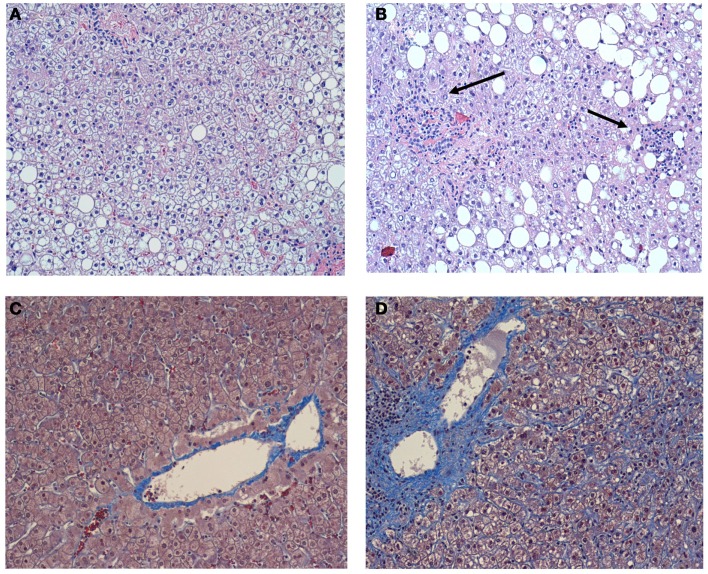
**(A)** A representative image of the liver without inflammation in the individual without OSA. Hematoxylin-eosin. X 100. Macrovesicular hepatic steatosis is evident, but inflammation is absent; **(B)** A representative image of the liver in the individual with OSA and severe nocturnal oxyhemoglobin desaturation. Hematoxylin-eosin.X 100. Macrovesicular hepatic steatosis is evident, lobular inflammation is present (arrows); **(C)** A representative image of the liver without pericellular fibrosis in the individual without OSA. Masson trichrome X 100; **(D)** A representative image of the liver in the individual with OSA and severe nocturnal oxyhemoglobin desaturation. Masson trichrome X 100. Prominent pericellular perisinusoidal fibrosis is present. Collagen depositions are stained in blue and have chicken-wire appearance. Reproduced with permission from Polotsky et al. (2009).

The gold standard of NAFLD diagnosis, staging, and prognosis is liver biopsy. The first case report of severe NASH with intralobular and periportal inflammation, hepatocyte ballooning, and pericellular fibrosis in a patient with obesity hypoventilation syndrome was published in 2002 (Saibara et al., 2002). Since that time a number of studies examining relationships between OSA and findings in liver biopsy have been conducted (Table 2). All of these studies are cross-sectional, because it is not feasible to obtain liver biopsy repeatedly due to ethical considerations. Studies, in which polysomnography (PSG) has not been performed could not be used for the reliable analysis of the OSA-NAFLD association and will not be further discussed. In the first PSG based study, Tanne et al. (2005) performed liver biopsy in a small subset of sleep clinic patients with elevated serum liver enzymes and found that subjects with severe OSA defined as the AHI > 50/h (*n* = 9), exhibited more severe liver steatosis, necrosis, and fibrosis than subjects with the AHI ≤ 50/h (*n* = 9). Other studies focused on the bariatric population taking an advantage of the availability of intra-operative liver biopsy. Kallwitz et al. (2007) performed a retrospective review of 101 patients who underwent gastric bypass surgery. Liver biopsy was performed only if the liver had abnormal appearance (enlargement, yellow discoloration, or nodularity). Both PSG and liver biopsy were performed in 85 patients. There was a trend toward a higher prevalence of OSA in patients with inflammation and fibrosis (11/15) compared with those with inflammation alone (22/48). There was no association between OSA and the degree of steatosis, presence of hepatitis, balloon degeneration, or fibrosis on liver biopsy. Similarly, Jouet et al. (2007) also reported the lack of association between OSA and histological markers of NASH. However, relationships between nocturnal hypoxemia and NASH had not been examined. Mishra et al. (2008) studied 101 bariatric patients with biopsy-proven NAFLD, all of whom had full PSG in a sleep laboratory. The lowest desaturation independently correlated with histological NASH. Both mean and lowest oxygen desaturation were independently associated with the presence of liver fibrosis, whereas there was no statistically significant relationships between the AHI and RDI (the respiratory disturbance index defined as a number of apneas, hypopneas, and respiratory effort related arousals per hour) and liver fibrosis. Polotsky et al. (2009) studied 90 consecutive bariatric patients, all of which underwent PSG, and reported the prevalence of OSA (the RDI > 5/h) of 81.1%. The analysis of liver biopsies (*n* = 20) showed that lobular inflammation, hepatocyte ballooning, the NAS, and liver fibrosis were associated with severe oxyhemoglobin desaturation (Figure 1), but not the RDI and this association was independent of BMI. Aron-Wisnewsky et al. (2012) performed continuous nocturnal pulse oximetry in 101 bariatric patients who underwent liver needle biopsy intra-operatively. Liver histology was compared in three tertiles of oxygen desaturation index (ODI), <6.7, 6.8–18.5, and >18.5 /h. The BMI was similar in all tertiles varying from 45.7 to 48.3 kg/m^2^, whereas patients with more severe OSA had higher prevalence of type 2 diabetes, dyslipidemia, and hypertension. There was a significant increase in liver steatosis, ballooning, lobular inflammation, NAS, and liver fibrosis with increased ODI. After the adjustment for type 2 diabetes, inflammation, age, and sex, the ODI remained independently associated with higher NAS and the severity of liver fibrosis. Notably, similarly to Polotsky et al. (2009), ALT and AST values were within the normal range in all patients, regardless of the severity of OSA and NASH. Thus, emerging evidence demonstrates that there is an association between the severity of hypoxic indexes in patients with OSA and the severity of NAFLD diagnosed by liver biopsy. The effect of CPAP treatment on NAFLD pathology has not been examined.

**Table 2:** **Cross-sectional studies that examined liver biopsies in patients with OSA**.

Study	Sample size	Outcome	Findings
Singh et al. (2005)	190 NAFLD patients (only steatosis on biopsy or CT/US in 116, biochemical NASH by ↑ALT or AST to 1.5 of normal in 74), including 50 confirmed by liver biopsy (18 steatosis, 32 NASH)	Modified Berlin Sleep Apnea Questionnaire for OSA. No PSG	Eighty-seven (46%) patients met criteria for OSA. The prevalence was similar in both biochemically and histologically defined steatosis and NASH.
Tanne et al. (2005)	163 patients suspected for OSA, 44 (27%) with severe OSA (AHI > 50/h), 84 (52%) with moderate OSA (AHI 10–50/h), 35 (21%) with no OSA (AHI < 10/h). Liver biopsy in 18 out of 32 with elevated liver enzymes	ALT, AST, GGT, α-GST. Steatosis, lobular necrosis, and fibrosis in liver biopsy	↑ALT and GGT in severe OSA compared to control. ↑in hepatic steatosis, lobular necrosis and fibrosis on liver biopsy in patients with severe OSA (*n* = 9) compared to moderate OSA or control (*n* = 9)
Kallwitz et al. (2007)	Records from 101 bariatric patients reviewed retrospectively. 85 patients had liver enzymes, sleep studies, and liver biopsy. 51% had OSA identified by an AHI ≥ 15/h	ALT, AST. Liver steatosis, inflammation, and fibrosis	↑ALT in OSA. ↑Prevalence of OSA in patients with inflammation + fibrosis. No association between OSA and AST, steatosis, hepatitis, balloon degeneration, or fibrosis
Jouet et al. (2007)	62 consecutive bariatric patients	ALT, AST, GGT, Liver biopsy	↑liver enzymes in OSA. No relationship between OSA and NASH on liver biopsy
Mishra et al. (2008)	101 bariatric patients, including 77 patients with NASH and 24 controls. 51% had OSA identified by an AHI > 5/h	PSG	↓ minimal and mean SpO_2_, in patients with NASH and liver fibrosis, ↑AHI in patients with NASH. Minimal SpO_2_ independently correlated with histological NASH
Polotsky et al. (2009)	90 consecutive bariatric patients. Liver biopsy in 20 subjects. All patients had PSG	ALT, AST, insulin, glucose. Liver biopsy	All patients had normal liver enzymes. Mean oxygen desaturation > 4.6% was associated with a 1.5 increase in HOMA. ↓SpO_2_ was associated with lobular inflammation, ballooning, and liver fibrosis, but not with steatosis
Daltro et al. (2010)	40 bariatric patients	PSG, fasting glucose, insulin, liver enzyme, liver biopsy	↑prevalence of OSA (80%), NAFD (82.5%), and NASH (80%). OSA was associated with insulin resistance but not with the severity of NASH
Aron-Wisnewsky et al. (2012)	101 bariatric patients (ODI < 6.7, *n* = 33; ODI 6.8–18.5, *n* = 34; ODI > 18.5, *n* = 34)	ALT, AST, glucose, insulin, HOMA, triglycerides Leptin, IL-6; Liver biopsy	No change in ALT, AST, leptin; ↑fasting blood glucose, insulin, IL-6. ↑steatosis, ballooning, inflammation, NAS, and fibrosis with severity of OSA

*α-glutathione-*S*-transferase*.

### OSA and hypoxic liver injury

Although the prevalence of NAFLD in patients with OSA remains unknown, clinical evidence suggests it might be a common phenomenon. In contrast, hypoxic liver injury with hyper-acute elevation of aminotransferases >1000, liver necrosis and coagulopathy has been described only in a handful of cases (Mathurin et al., 1995; Henrion et al., 1997, 2003; Trakada et al., 2004). Patients with OSA-induced liver injury uniformly had obesity hypoventilation syndrome with daytime hypoxemia, and sleep disordered breathing in these cases was characterized by severe hypoxemia with oxyhemoglobin saturation below 70% for prolonged periods of time. Nevertheless, current clinical evidence demonstrates that liver pathology in OSA by enlarge is not a result of simple liver tissue hypoxia.

## IH and NAFLD: Experimental Evidence

A mouse model of IH mimicking oxyhemoglobin desaturations in patients with OSA has been utilized with a FiO_2_ fluctuating between 20.9 and 5% 60 times/h during the 12 h light phase (9am to 9pm) when 70% of mouse sleep occurs (Polotsky et al., 2006). O_2_ swings resulted in reciprocal changes in oxyhemoglobin saturation (SpO_2_) from 95–98% to mid 60%–low 70% (Reinke et al., 2011a,b). Lean C57BL/6J mice exposed to such a regimen did not immediately develop liver injury. In fact, lipid peroxidation increased in the liver only after 4 weeks of exposure, but serum aminotransferases remained within the normal range (Jun et al., 2008; Drager et al., 2011). Only after 12 weeks of exposure, lean C57BL/6J mice exhibited a mild increase in serum ALT to ∼200 U/L and exhibited moderate hepatocellular swelling without steatosis or cellular apoptosis (Savransky et al., 2007c). The presence of obesity dramatically exacerbated effects of IH. In C57BL/6J mice with diet-induced obesity (DIO), IH for 4 weeks increased the degree of hepatic steatosis, induced lipid peroxidation on the liver, elevated ALT, AST, and AP levels and exacerbated insulin resistance, a hallmark of NASH (Drager et al., 2011). In DIO mice, but not in lean mice, IH increased RNA and protein levels of pro-inflammatory cytokines TNF-α and MIP-2 in the liver suggesting the progression of hepatic steatosis to steatohepatitis (Drager et al., 2011). IH for 6 months converted diet-induced hepatic steatosis to NASH with liver fibrosis (Savransky et al., 2007a). Thus, experimental evidence suggests that IH *per se* is not sufficient to cause NAFLD in the mouse model. IH and obesity interact to exacerbate hepatic steatosis converting it to steatohepatitis.

## Mechanisms of NAFLD and NASH in OSA

**Figure 2:**
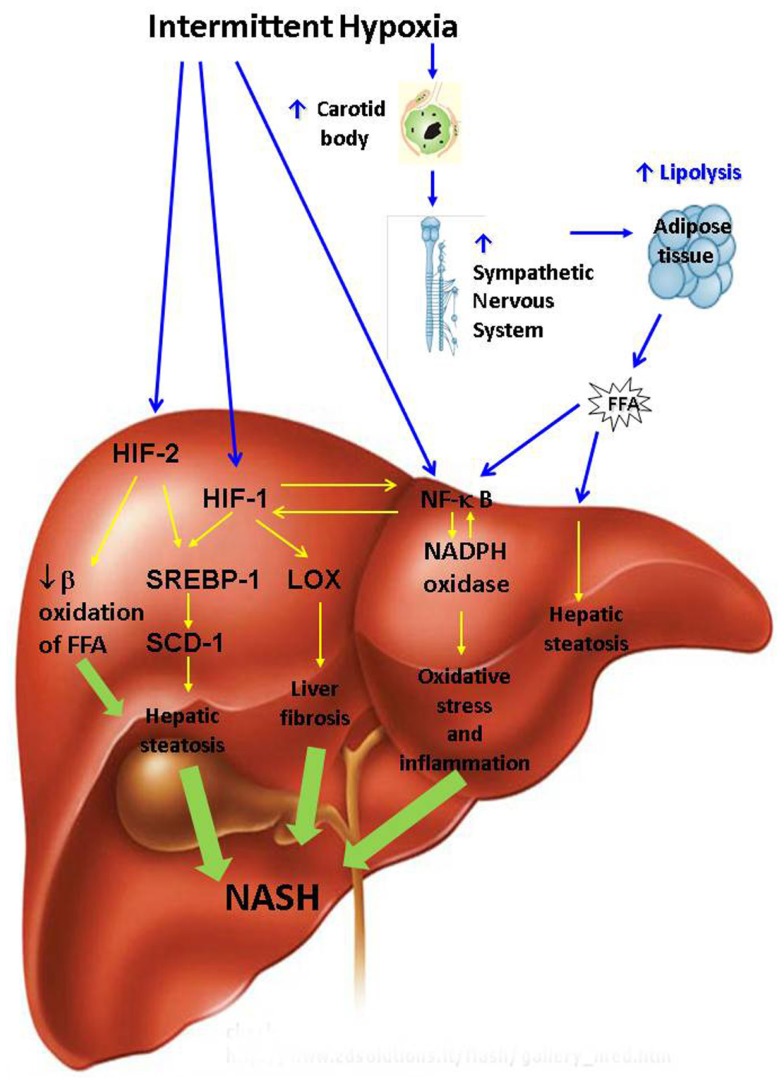
**Putative pathways leading to non-alcoholic steatohepatitis (NASH) during intermittent hypoxia of obstructive sleep apnea**. FFA, free fatty acids; HIF, hypoxia inducible factor; LOX, lysyl oxidase; NADPH, nicotinamide adenine dinucleotide phosphate (NADPH); NF-κB, nuclear factor kappa B; SCD, stearoyl coenzyme A desaturase; SREBP, sterol regulatory element binding protein.

Putative mechanisms by which OSA may promote the development and progression of NAFD are depicted in Figure 2. OSA encompasses several physiological mechanisms which may predispose to NAFLD including negative intra-thoracic pressure swings, sleep fragmentation, hypercapnea, and IH. However, the relationship between NAFLD and IH is the only one which has been studied and will be further discussed. We will first review mechanisms by which IH may lead to hepatic steatosis.

### IH and hepatic steatosis

Intermittent hypoxia may exacerbate hepatic steatosis via systemic and tissue specific mechanisms. Systemic mechanisms are likely mediated via the sympathetic nervous system (SNS). Obesity and insulin resistance are characterized by increased adipocyte mass and increased hormone-sensitive lipase activity, which leads to up-regulation of lipolysis and increased uptake of FFA by the liver (Browning and Horton, 2004). SNS is a major regulator of lipolysis (Jaworski et al., 2007; Bickel et al., 2009; Lafontan and Langin, 2009; Zechner et al., 2009). Human OSA and IH in rodents activate SNS and increase circulating catecholamine levels (Somers et al., 1989, 1995; Carlson et al., 1993; Dimsdale et al., 1995; Fletcher et al., 1995; Hedner et al., 1995; Bao et al., 1997; Narkiewicz et al., 1998, 1999). Activation of the SNS during IH occurs through the hypoxic chemo-reflex in the carotid body and ablation of the carotid sinus nerve prevents IH-induced hypertension (Fletcher et al., 1992a,b; Fletcher, 2001; Prabhakar et al., 2007; Prabhakar and Kumar, 2010). We and others have recently shown that sleep apnea raises circulating FFA levels in proportion to the severity of hypoxia (Barcelo et al., 2011; Jun et al., 2011) suggesting that IH leads to exuberant lipolysis in adipose tissue. The FFA influx into the liver may induce insulin resistance and triglyceride biosynthesis leading to hepatic steatosis (Delarue and Magnan, 2007; Guilherme et al., 2008; Jocken and Blaak, 2008; Neuschwander-Tetri, 2010; Tilg and Moschen, 2010). In addition, high levels of insulin in obese individuals may up-regulate hepatic lipid biosynthesis *de novo* by activating SREBP-1c (Foretz et al., 1999; Shimomura et al., 1999b) and a SREBP-1-regulated enzyme of triglyceride biosynthesis SCD-1 (Cohen et al., 2002; Biddinger et al., 2005). Exposure to IH for 5 days to 12 weeks uniformly activated the SREBP-1c axis and downstream SCD-1 in both lean and obese mice inducing triglyceride accumulation in the liver, which was more pronounced in DIO and in mice with leptin-deficient obesity (Li et al., 2005a,b, 2007; Savransky et al., 2007b; Drager et al., 2011). It is conceivable that IH induces hepatic steatosis by up-regulating hypoxia inducible factors 1 and 2, masters-regulators of metabolic responses to hypoxia composed of constitutively expressed HIF-1β and O_2_ responsive 1α and 2α subunits respectively (Semenza, 2007; Semenza and Prabhakar, 2007; Majmundar et al., 2010). Obesity is associated with liver tissue hypoxia, which may be further exacerbated by IH of OSA (Reinke et al., 2011a) resulting in HIF activation. Partial global deficiency of HIF-1 α of hypoxia inducible factor 1α abolished SREBP-1 and SCD-1 up-regulation and prevented triglyceride accumulation in the liver during IH (Li et al., 2006). However, HIF-1 is implicated in carotid body activation during IH and the HIF-1α effect may be mediated systemically via SNS rather than directly on the liver (Peng et al., 2006). Additional insight into the potential role of HIFs in the pathogenesis of hepatic steatosis during IH has been obtained from mice with deficiency of Von Hippel-Lindau (VHL) tumor suppressive protein in the liver. VHL is required for HIF degradation, therefore VHL mice exhibit a phenotype of constitutive HIF up-regulation (Haase et al., 2001; Rankin et al., 2005). VHL deficient mice have severe hepatic steatosis which was prevented by HIF-2α but not HIF-1α knockout suggesting that HIF-2 contributes to the pathogenesis of hepatic steatosis (Rankin et al., 2009; Qu et al., 2011). In the VHL model HIF-2α up-regulated lipid biosynthetic genes and down-regulated genes of β-oxidation. However, applicability of findings in the VHL KO model to hepatic steatosis induced by IH is uncertain. It remains unknown whether up-regulation of lipogenic genes during IH occurs due to direct activation of HIFs in the liver or predominantly due to the FFA flux from adipose tissue.

### IH and NASH

Putative mechanisms of NAFLD progression to NASH are depicted in Figure 2. FFA flux to the liver induced by IH (Drager et al., 2011; Jun et al., 2011) may up-regulate IκB kinase β resulting in phosphorylation and degradation of IκB followed by activation of NF-κB and ensuing synthesis of pro-inflammatory cytokines tumor necrosis factor alpha (TNF-α), interleukin 6 (IL-6), macrophage inflammatory protein 2 (MIP-2) and others (Kim et al., 2001; Sinha et al., 2004; Boden et al., 2005; Cai et al., 2005). We have previously reported activation of NF-κB in the liver by IH (Savransky et al., 2007c). In the presence of obesity and hepatic steatosis, IH increased mRNA and protein levels of pro-inflammatory cytokines TNF-α, IL-1, MIP-2 in liver tissue (Savransky et al., 2007a,b; Drager et al., 2011). It is conceivable that IH and obesity interact to induce NASH via accelerated adipose tissue lipolysis. However, this hypothesis is yet to be proven in rodent experiments and clinical studies with lipolysis inhibitors.

Intermittent hypoxia may also lead to NASH by up-regulating reactive oxygen species (ROS) generation via NADPH oxidase system (Jun et al., 2008). NADPH oxidase stimulates hepatic stellate cells to produce collagen via angiotensin II leading to liver fibrosis (Bataller et al., 2003; Novitskiy et al., 2006). NADPH oxidase can activate NF-κB (Brar et al., 2003). Reactive oxygen may also directly stimulate NF-κB (Li and Karin, 1999) inducing downstream inflammatory pathways (Ben-Neriah and Karin, 2011). There is a cross-talk between NF-kB and HIF-1α, which mutually activate each other (Walmsley et al., 2005; Scortegagna et al., 2008; Taylor and Cummins, 2009). NF-κB and HIF-1 up-regulate synthesis of collagen, while HIF-1 also induces transcription of lysyl oxidase resulting in cross-linking of collagen (Novitskiy et al., 2004; Manalo et al., 2005; Copple et al., 2009, 2011; Haase, 2009). Paradoxically, HIF-1 may be protective in ischemia-reperfusion liver injury by preventing hepatocyte apoptosis (Schneider et al., 2010; Lehwald et al., 2011; Nath and Szabo, 2012). However, NASH in OSA is a fundamentally different process from ischemia-reperfusion injury observed in “shock liver,” which is mediated by the intricate interplay of systemic and tissue specific processes described above and in Figure 2. We have previously demonstrated that IH causes hepatic inflammation and fibrosis in mice with diet-induced hepatic steatosis (Savransky et al., 2007a; Drager et al., 2011). We have also shown that IH causes more severe liver hypoxia in obese mice (Reinke et al., 2011a). It is conceivable that severe liver hypoxia in obese individuals with severe OSA induces NF-kB and HIF-1 in the liver resulting in steatohepatitis and liver fibrosis. However, this hypothesis is yet to be tested.

## Conclusion

Obstructive sleep apnea and NAFLD are common complications of obesity. The causality of NAFLD has been linked to obesity, but mechanisms of NAFLD progression to NASH are unknown and there is no effective treatment. Emerging evidence suggests that OSA may contribute to the development and progression of NAFLD to NASH. However, many questions remain unanswered. First, the causal link between OSA and NAFLD has not been established. Prospective clinical studies and a randomized placebo controlled clinical trials of CPAP in patients with NAFLD and NASH will have to be conducted. Second, mechanisms by which OSA and IH may lead to NAFLD and NASH remain unclear. The mouse model of IH will allow to explore such mechanisms utilizing targeted pharmacological interventions (sympathetic blockade, antioxidants, and lipolysis inhibitors) and transgenic animals with liver specific knockouts of gene candidates (HIFs, NADPH oxidase, NF-κB).

## Conflict of Interest Statement

Dr. Mirrakhimov declared his research was conducted in the absence of any commercial or financial relationships that could be construed as a potential conflict of interest. Dr. Polotsky is supported by the NIH grant R01 HL080105 and by ResMed Foundation grant 111481.
